# Supplementary material for: Dufour’s gland analysis reveals caste and physiology specific signals in Bombus impatiens
Source: Sci Rep. 2021 Feb 2;11:2821. doi: 10.1038/s41598-021-82366-2 (PMC7854627; doi:10.1038/s41598-021-82366-2)
Supplement: Supplementary file 2 — Supplementary Information 2. [file 41598_2021_82366_MOESM2_ESM.pdf]

Dufour's gland analysis reveals caste and physiology specific signals in *Bombus impatiens*

Nathan T. Derstine[1], Gabriel Villar[1,] Margarita Orlova[1], Abraham Hefetz[2,3], Jocelyn Millar[4], Etya Amsalem[1]

[1]Department of Entomology, Center for Chemical Ecology, Center for Pollinator Research, Huck Institutes of the Life Sciences, Pennsylvania State University, University Park, PA 16802 U.S.A.

[2]School of Zoology, George S. Wise Faculty of Life Sciences, Tel Aviv University, Israel

[3]School of Marine Sciences, Ruppin Academic Center, Israel

[4]Departments of Entomology and Chemistry, University of California Riverside, CA 92521

Supplementary Information

Figure S1 – Diagram of the two-choice olfactometer design. Olfactometers were fashioned from petri dishes (150 × 15 mm) where two equidistant holes (2 cm diameter) led to small plastic cups that held treatment or control stimuli on a glass slide. A 3 cm section of wide plastic straw was glued onto the bottom of the hole and extended into the cup, preventing re-entry into the main arena. Bees could not contact the glass slides unless they fully entered the cup, having fallen from the end of the plastic straw. Drawn in Microsoft Powerpoint for Mac version 16.62.

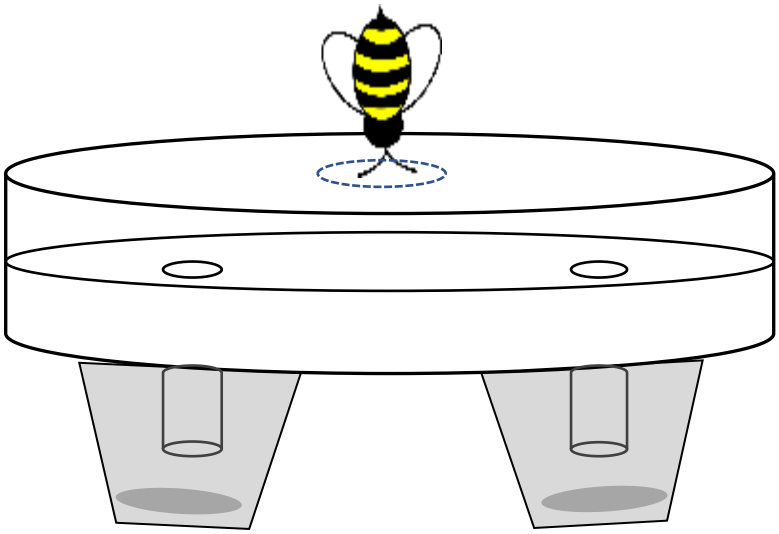

Figure S2 – Representative GC-FID chromatograms of *B. impatiens* workers, gynes and queens. Number labels correspond to compounds identified from hexane extracts of the Dufour's gland used in statistical comparisons. **1** – β-springene, **2** – springene isomer I, **3** – springene isomer II, **4** – eicosane IS (C_{20}), **5** – heneicosane (C_{21}), **6** – dodecyl-octanoate, **7** – docosane (C_{22}), **8** – tricosene ($C_{23:1}$), **9** – tricosane (C_{23}), **10** – dodecyl-decanoate, **11** – tetracosane (C_{24}), **12** – pentacosane ($C_{25:1}$), **13** - C_{25}, **14** – ester complex 1 (dodecyl-decanoate), **15** – heptacosene $C_{27:1}$, **16** – heptacosane C_{27}, **17** – ester complex 2 (hexadecyl decanoate), **18** – nonacosene I ($C_{29:1}$), **19** - nonacosene II ($C_{29:1}$), **20** – nonacosane (C_{29}), **21** – ester complex 3 dodecyl-9Z-hexadecenoate, **22** – hentriacontene I ($C_{31:1}$), **23** - hentriacontene II ($C_{31:1}$), **24** – hentriacontene (C_{31}), **25** - dodecyl-octadecenoate, **26** - ester complex 4 (octadecenyl tetradecanoate), **27** – ester complex 5 (octadecenyl hexadecanoate), **28** – terpene ester I, **29** – terpene ester II.

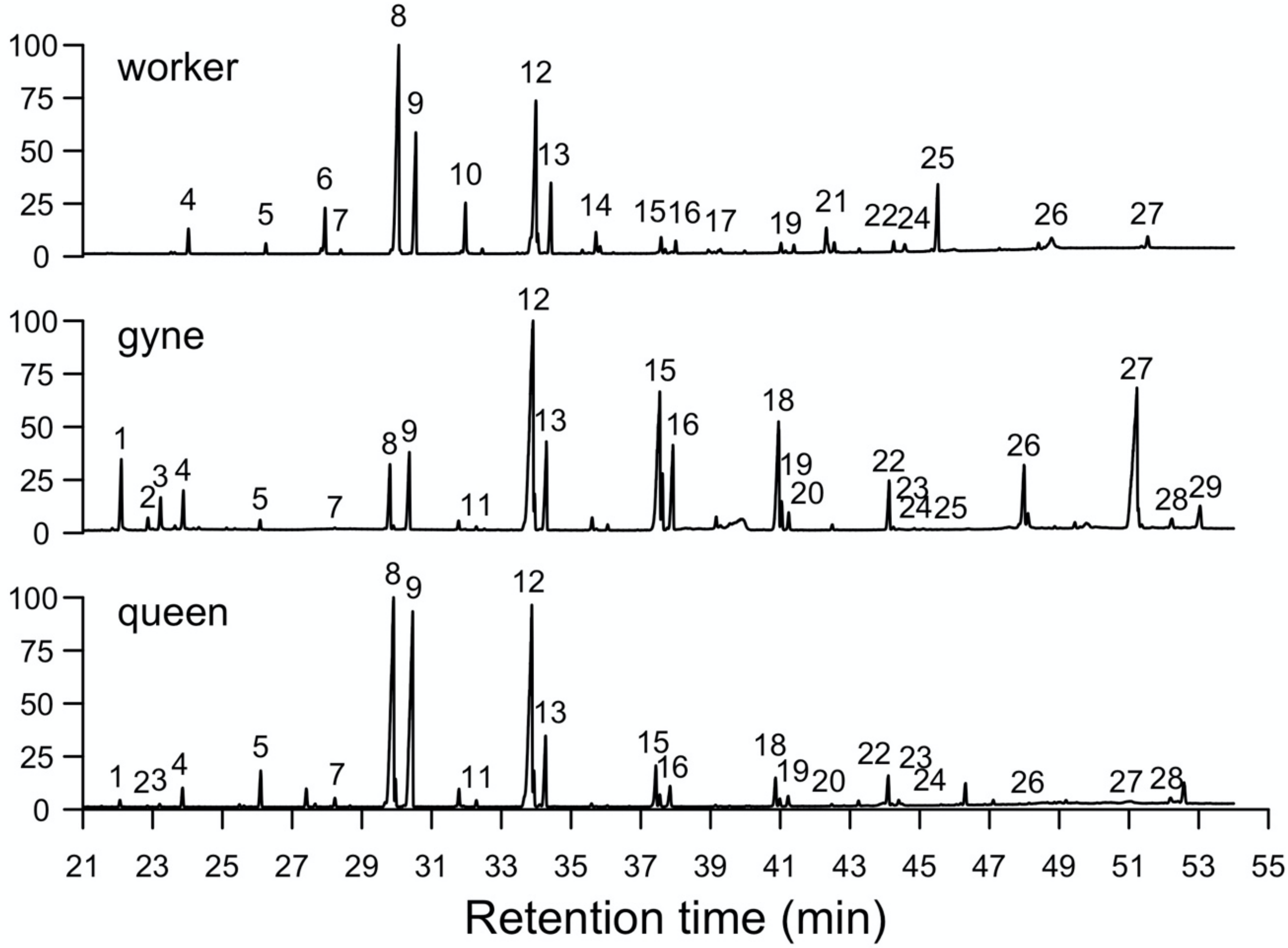

Figure S3 – Linear discriminant analyses on Z-transformed relative peak areas of Dufour's gland extracts of workers in three social conditions: queenless (QL), queenless brood-less (QLBL), and queenright (QR). Each point is the value from an individual bee. Panels a-d show the clustering generated by plotting the first two linear discriminant functions using esters, alkanes, alkenes, or all compounds.

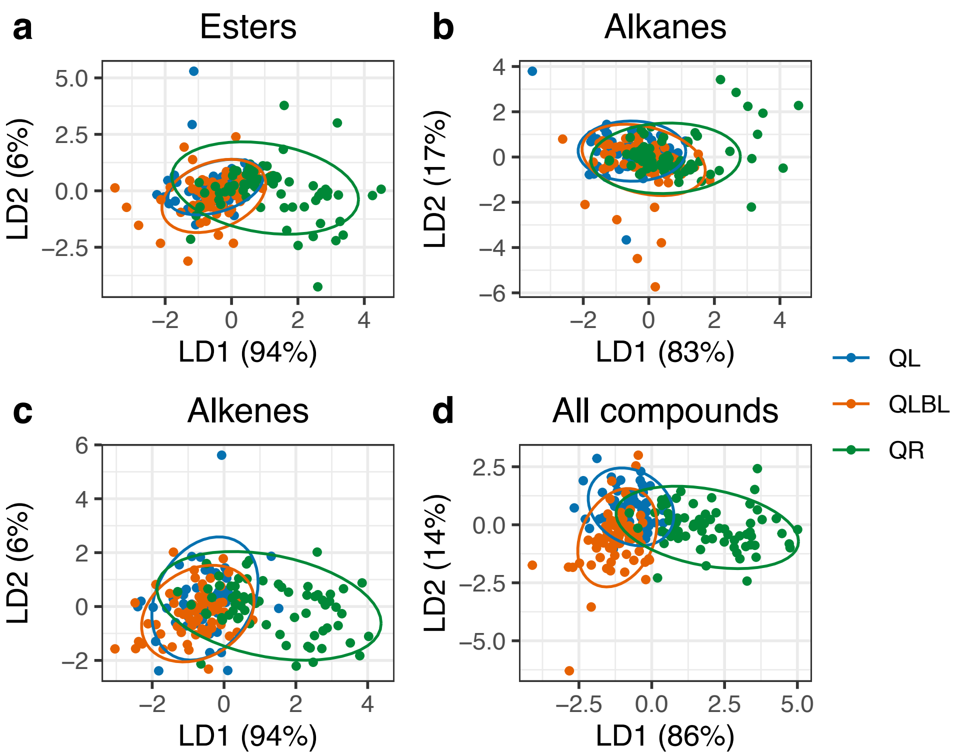

Figure S4 – Mean amount (µg) of the Dufour's gland chemical class "hydrocarbon" in 1-14 day old *B. impatiens* workers that were kept under three social condition treatments: queenless (QL), queenless and broodless (QLBL), or queenright (QR), shown as blue, orange, or green, respectively (n = 10 per treatment per day). The class hydrocarbon is separated into "alkane" and "alkene".

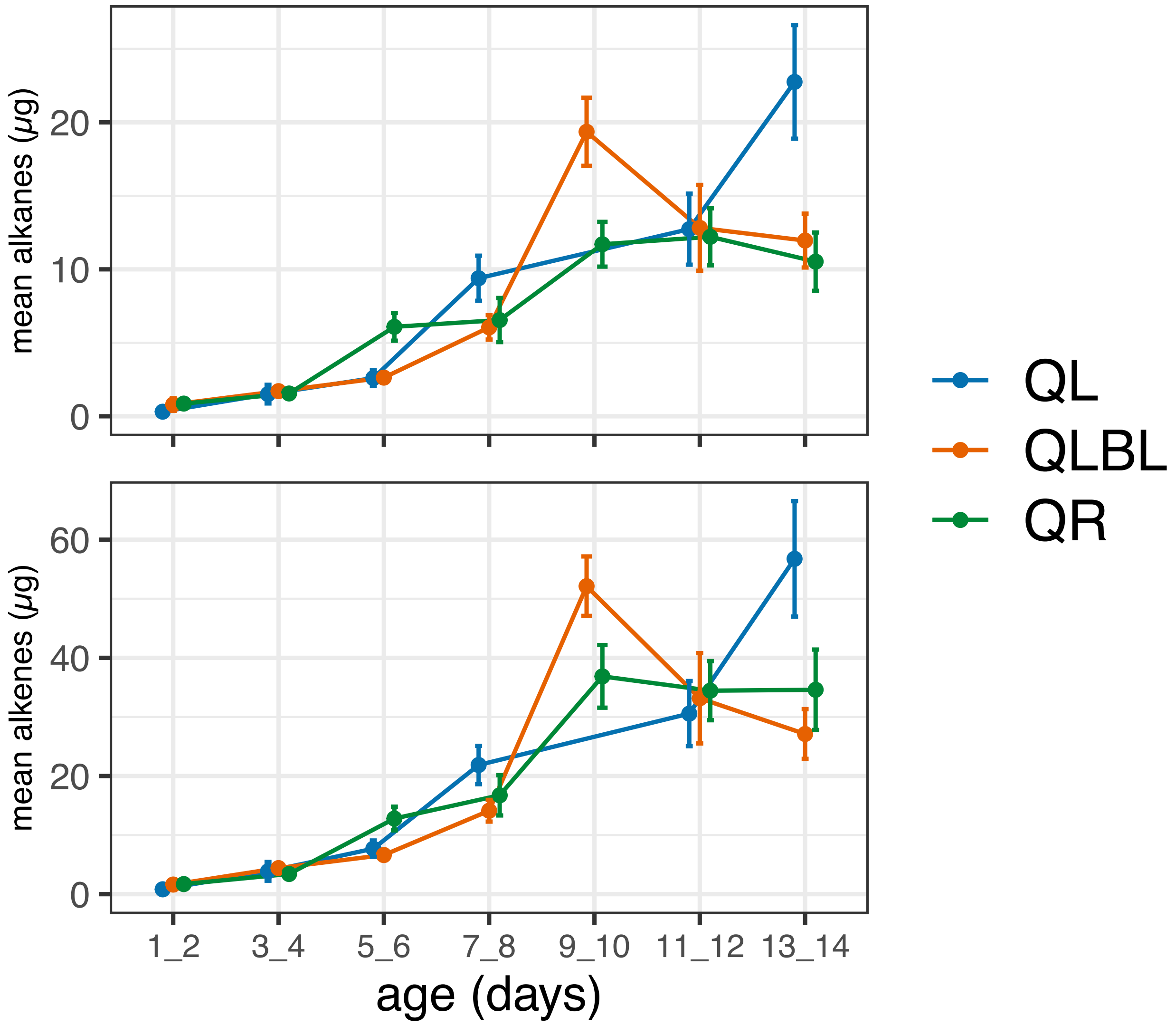

Table S1 - Summary of sample sizes per treatment and colony for *B. impatiens* workers, gynes, and queens used in chemical analyses of the Dufour's gland, olfactometer and EAG experiments in the current study. Colony was included in statistical models as a random factor.

Experiment	Caste/treatment	Total sample size	Number of colonies
Chemical analysis of DG*	QL workers	60	7
	QLBL workers	70	
	QR workers	69	
	Gynes	20	3
	Queens	20	20
Olfactometer experiment**	QR workers	47	11
	QLBL workers	40	
EAG experiment	QR workers	13	3
	QLBL workers	16	
Total		315 workers<br>40 gynes/queens	38 different colonies

* The total sample size for workers does not include outliers that were excluded from the chemical analysis (n=11, see Results for an explanation)

** The total sample size does not include non-responsive workers (n=35, see Results for an explanation)

Table S2 – Relative abundances (percent of total secretion) and absolute amounts (μg) of Dufour's glands compounds used in discriminant analyses, for mated queens (n = 20), virgin gynes (n = 20), and workers (n = 199) of all ages. Data are presented as means ± S.E.M. Kovat's retention indices, retention times, and diagnostic ions are also provided.

Peak	Compound ID	Class	Retention Index (DB-5MS)	Retention time (DB-5MS)	Diagnostic Ions[a]	Queen relative amount	Gyne relative amount	Worker relative amount	Queen amount (μg)	Gyne amount (μg)	Worker amount (μg)
1	β-springene	diterpene	1920	22.10	41, 55, 69, 81, **272**	1.3 ± 0.33	3.2 ± 0.19	0 ± 0	1.82 ± 0.72	2.94 ± 0.4	-
2	Springene isomer I	diterpene	1953	22.86	41, 55, 69, 81, **272**	0.2 ± 0.05	0.5 ± 0.03	0 ± 0	0.29 ± 0.11	0.43 ± 0.06	-
3	Springene isomer II	diterpene	1968	23.23	41, 55, 69, 81, **272**	0.5 ± 0.14	1.3 ± 0.07	0 ± 0	0.71 ± 0.28	1.21 ± 0.16	-
4	C_{20} (internal standard) – Eicosane	alkane	2000	23.23	**282**	NA	NA	NA	NA	NA	NA
5	C_{21} – Heneicosane	alkane	2100	26.08	**296**	1.0 ± 0.11	0.7 ± 0.06	0.5 ± 0.01	1.51 ± 0.46	0.64 ± 0.08	0.18 ± 0.01
6	Dodecyl octanoate	ester	2173	27.94	127,145, 168, **312**	-	-	1.0 ± 0.07	-	-	0.36 ± 0.04
7	C_{22} – Docosane	alkane	2200	28.23	**310**	0.3 ± 0.02	0.1 ± 0.01	0.2 ± 0.01	0.41 ± 0.11	0.1 ± 0.02	0.08 ± 0.01
8	$C_{23:1}$ – Tricosene (two isomers)	alkene	2275	29.81	**322**	23.1 ± 1.57	7.1 ± 0.52	22.5 ± 0.41	31.54 ± 8.53	6.46 ± 0.81	7.88 ± 0.57
9	C_{23} – Tricosane	alkane	2300	30.36	**324**	15.6 ± 0.78	6.0 ± 0.26	10.6 ± 0.17	22.73 ± 6.37	5.19 ± 0.57	3.79 ± 0.28
10	Dodecyl-decanoate	ester	2369	31.97	155,173, 168, **340**	-	-	2.6 ± 0.08	-	-	0.89 ± 0.07
11	C_{24} – Tetracosane	alkane	2400	32.28	**338**	0.4 ± 0.02	0.2 ± 0.01	0.4 ± 0.01	0.55 ± 0.15	0.13 ± 0.01	0.14 ± 0.01
12	$C_{25:1}$ – Pentacosene (two isomers)	alkene	2477	33.91	**350**	31.8 ± 1.14	28.2 ± 0.84	27.7 ± 0.36	51.35 ± 15.34	24.43 ± 2.61	9.46 ± 0.7
13	C_{25} – Pentacosane	alkane	2500	34.40	**352**	6.5 ± 0.41	4.2 ± 0.18	8.1 ± 0.15	10.42 ± 3.31	3.43 ± 0.29	2.59 ± 0.19
14	Dodecyl dodecanoate (ester complex 1*)	ester	2567	35.71	183,201, 168, **368**	-	-	1.8 ± 0.08	-	-	0.66 ± 0.07
15	$C_{27:1}$ – Heptacosene	alkene	2673	37.54	**378**	5.0 ± 0.69	12.6 ± 0.3	3.4 ± 0.1	14.13 ± 6.61	10.41 ± 0.96	1.24 ± 0.12
16	C_{27} – Heptacosane	alkane	2700	38.2	**380**	1.7 ± 0.25	3.5 ± 0.23	1.4 ± 0.04	4.71 ± 2.23	2.78 ± 0.23	0.5 ± 0.05
17	Hexadecyl decanoate (ester complex 2*)	ester	2769	39.28	155,173, 224, **396**	-	-	1.5 ± 0.13	-	-	0.55 ± 0.06
18	$C_{29:1}$ – Nonacosene I	alkene	2874	40.94	**392**	2.7 ± 0.39	5.4 ± 0.53	1.2 ± 0.04	7.02 ± 3.47	3.98 ± 0.37	0.56 ± 0.06
19	$C_{29:1}$ – Nonacosene II	alkene	2881	41.45	**392**	1.0 ± 0.25	1.9 ± 0.4	0.5 ± 0.02	3.56 ± 2.04	1.9 ± 0.64	0.18 ± 0.02
20	C_{29} – Nonacosane	alkane	2900	41.79	**394**	1.8 ± 0.31	0.9 ± 0.12	0.8 ± 0.02	2.05 ± 0.65	0.67 ± 0.09	0.28 ± 0.02
21	Dodecyl hexadecenoate (ester complex 3*)	ester	2951	42.50	194, 236, 255, **422**	0.1 ± 0.02	-	3.1 ± 0.16	0.03 ± 0.01	0 ± 0	1.37 ± 0.21

22	$C_{31:1}$ – Hentriacontene I	alkene	3075	44.12	**434**	3.0 ± 0.45	2.1 ± 0.13	1.2 ± 0.06	5.59 ± 3.12	1.67 ± 0.16	0.65 ± 0.08
23	$C_{31:1}$ – Hentriacontene II	alkene	3083	44.80	**434**	0.4 ± 0.24	0.1 ± 0.01	0.2 ± 0.02	1.92 ± 1.66	0.07 ± 0.01	0.12 ± 0.02
24	C_{31} – Hentriacontane	alkane	3100	44.90	**436**	1.7 ± 0.44	0.03 ± 0.01	0.4 ± 0.02	0.77 ± 0.13	0.02 ± 0.01	0.15 ± 0.01
25	Dodecyl octadecenoate	ester	3155	45.52	180, 222, 264, 283, **450**	0.04 ± 0.01	0.01 ± 0.01	5.1 ± 0.29	0.03 ± 0.01	-	1.59 ± 0.14
26	Octadecenyl tetradecanoate (ester complex 4*)	ester	3333	47.99	209, 227, 250, **478**	0.3 ± 0.1	5.4 ± 0.24	2.4 ± 0.16	0.53 ± 0.32	4.4 ± 0.37	1.16 ± 0.17
27	Octadecenyl hexadecenoate (ester complex 5*)	ester	3539	51.23	237, 255, **506**	0.5 ± 0.25	13.2 ± 1.05	3.3 ± 0.27	1.7 ± 1.02	10.38 ± 0.97	1.12 ± 0.12
28	Terpene ester I	ester	3601	52.23	41, 55, 69, 81, 136	1.0 ± 0.21	1.2 ± 0.18	-	0.82 ± 0.31	1.17 ± 0.21	-
29	Terpene ester II	ester	3643	53.20	41, 55, 69, 81, 135, 272	0.1 ± 0.04	2.2 ± 0.26	-	0.18 ± 0.13	2.13 ± 0.35	-

* Ester complexes consisted of multiple often overlapping or co-eluting esters that were treated as a single variable. The most prevalent compound in the complex is given as the name. Other compounds in a given complex are esters with the same total number of carbons as the predominate compound, but different chain lengths of the acid or alcohol portion.

[a] Mass ion is given in bold except for compounds 28 and 29, for which the mass ion is unclear and possibly beyond the high mass scan threshold (550).
